# One new species of the genus *Sinopoda* from Hubei Province, with description of the male of *Sinopoda angulata* (Araneae, Sparassidae)

**DOI:** 10.3897/BDJ.8.e55377

**Published:** 2020-09-03

**Authors:** Yang Zhu, Yang Zhong, Tingbang Yang

**Affiliations:** 1 The State Key Laboratory of Biocatalysis and Enzyme Engineering of China, Centre for Behavioural Ecology and Evolution, College of Life Sciences, Hubei University, Wuhan 430062, Hubei, China The State Key Laboratory of Biocatalysis and Enzyme Engineering of China, Centre for Behavioural Ecology and Evolution, College of Life Sciences, Hubei University Wuhan 430062, Hubei China; 2 Hubei Key Laboratory of Radiation Chemistry and Functional Materials, School of Nuclear Technology and Chemistry & Biology, Hubei University of Science and Technology, Xianning 437100, Hubei, China Hubei Key Laboratory of Radiation Chemistry and Functional Materials, School of Nuclear Technology and Chemistry & Biology, Hubei University of Science and Technology Xianning 437100, Hubei China; 3 Institute of Ecology, Key Laboratory of Southwest China Wildlife Resources Conservation (Ministry of Education), China West Normal University, Nanchong 637009, China Institute of Ecology, Key Laboratory of Southwest China Wildlife Resources Conservation (Ministry of Education), China West Normal University Nanchong 637009 China

**Keywords:** biodiversity, huntsman spiders, China, taxonomy

## Abstract

**Background:**

In the past year, Prof. Jian Chen conducted several spider collections in Hubei Province. Almost 1000 spiders were collected. After diagnosis, two of them were found to belong to the genus *Sinopoda* Jäger, 1999.

**New information:**

Two *Sinopoda* Jäger, 1999 species, both from Hubei Province, including one new species, are treated in the current paper: *S. angulata* Jäger, Gao & Fei, 2002 and *S. yichangensis* **sp. n.** (♂). The male of *S. angulata* is described for the first time from Enshi Tujia and Miao Autonomous Prefecture, Hubei Province. New geographic records are provided as well as photos of the copulatory organs and habitus.

## Introduction

The genus *Sinopoda* was established by Jäger (1999), with *Sinopoda forcipata* (Karsch, 1881) as its type species. Currently, *Sinopoda* is the fourth most speciose genus of the subfamily Heteropodinae Thorell, 1873, and includes 126 species (Bertkau 1872, Thorell 1873, Plantnick 2020). Of these, a total of 41 species were collected from caves (Plantnick 2020). The species of the genus *Sinopoda* are small to large spiders whose body length ranges from 3 to 26 mm, with much longer legs relative to their body (Jäger 1999). Members of this genus are known from South-, East- and Southeast-Asia: Brunei, China, India, Indonesia, Japan, Korea, Laos, Malaysia, Thailand and Vietnam (Grall and Jäger 2020). From China, 65 species are known; among them, 18 species are only known from females and 4 from males (Plantnick 2020). The species *S. angulata* was first described based on a female specimen from Shennongjia National Nature Reserve, Hubei Province. Recently, the authors examined specimens collected from Hubei Province and found that two females and two males seemed to belong to this species. One new species of this genus is also described in this paper.

## Materials and methods

Specimens were examined and measured with a Leica M205C stereomicroscope. Points arising from the tegular appendages are listed as clock-positions from the left bulb in ventral view. Male palps were examined after dissection and detached from the spiders’ bodies and the epigynes were examined and illustrated after dissection. Epigynes were removed and cleared in warm lactic acid before illustration. All photographs were taken with a Leica DFC450 digital camera attached to a Leica M205C stereomicroscope, with 10–20 photographs taken in different focal planes and combined using image stacking software (Leica LAS). Photographic images were edited using Adobe Photoshop CC 2015. Left palps are illustrated. Most hairs and macrosetae are omitted in the palp drawings. All specimens are deposited in the Centre for Behavioural Ecology and Evolution, College of Life Sciences, Hubei University, Wuhan, China (**CBEE**).

Leg measurements are shown as: total length (femur, patella, tibia, metatarsus, tarsus). The number of spines is listed for each segment in the following order: prolateral, dorsal, retrolateral, ventral (in femora and patellae, ventral spines are absent and the fourth digit is omitted in the spination formula). Abbreviations used in the text and figures are given below:

**ALE**—anterior lateral eyes;

**AME**—anterior median eyes;

**AW**—anterior width of prosoma;

**C**—conductor;

**CH**—clypeus height;

**dRTA**—dorsal branch of RTA;

**E**—embolus;

**EA**—embolic apophysis;

**FD**—fertilisation duct;

**FE**—femur;

**GA**—glandular appendage;

**LL**—lateral lobes;

**LS**—lobal septum;

**Mt**—metatarsus;

**OL**—opisthosoma length;

**OW**—opisthosoma width;

**Pa**—patella;

**PL**—prosoma length;

**PLE**—posterior lateral eyes;

**PME**—posterior median eyes;

**Pp**—palp or palpus;

**PP**—posterior part of spermathecae;

**PW**—prosoma width;

**RTA**—retrolateral tibial apophysis;

**SP**—spermophor;

**ST**—subtegulum;

**T**—tegulum;

**Ta**—tarsus;

**Ti**—tibia. I, II, III, IV—legs I to IV;

**vRTA**—ventral branch of RTA.

## Taxon treatments

### Sinopoda angulata

Jäger, Gao & Fei, 2002

FDD57396-9AE9-561D-A8D6-46F77AEB055A

#### Materials

- **Type status:** Other material. **Occurrence:** recordedBy: Jian Chen; individualCount: 4; sex: 2 females and 2 males; lifeStage: adult; preparations: in ethyl alcohol; **Taxon:** scientificName: *Sinopoda angulata* Jäger, Gao & Fei, 2002; order: Araneae; family: Sparassdiae; genus: Sinopoda; specificEpithet: angulata; scientificNameAuthorship: Jäger, Gao & Fei, 2002; **Location:** country: China; countryCode: CHN; stateProvince: Hubei Province; county: Badong; locality: Jingsihou National Nature Reserve; decimalLatitude: 31.33; decimalLongitude: 110.42; **Identification:** identifiedBy: Jian Chen; dateIdentified: August 2019; **Event:** samplingProtocol: by hand; year: 2019; month: 8; day: 24

#### Description

***Male*** (Fig. 3). PL 6.5, PW 5.3, AW 2.8, OL 6.7, OW 4.2. Eyes: AME 0.34, ALE 0.43, PME 0.39, PLE 0.48, AME–AME 0.23, AME–ALE 0.07, PME–PME 0.28, PME–PLE 0.63, AME–PME 0.40, ALE–PLE 0.46, CH AME 0.16, CH ALE 0.23. Spination: Palp: 131, 101, 2121; Fe: I–III 323, IV 321; Pa: I–IV 101; Ti: I 2026, III–IV 2126; Mt: I–II 1014, III–IV 3036. Measurements of palp and legs: Palp 11.0 (3.3, 1.4, 2.2, –, 4.1), I 36.9 (9.5, 3.2, 10.2, 10.9, 3.1), II 40.2 (10.3, 2.8, 11.6, 12.3, 3.2), III 30.6 (8.2, 2.2, 8.7, 8.9, 2.6), IV 33.4 (8.8, 2.3, 9.0, 10.2, 3.1). Leg formula: II-I-IV-III. Cheliceral furrow with three anterior and four posterior teeth, with 28 denticles. Dorsal prosoma reddish to yellowish-brown, posterior margins dark, with distinct fovea and shallow radial furrows. Chelicerae deep reddish-brown. Sternum yellowish-brown, with margin deep brown. Gnathocoxae and labium deep yellowish-brown, with margin deep brown. Legs yellowish-brown, with dark spots. Dorsal opisthosoma yellowish-brown with distinct bright patch in posterior half. Ventral opisthosoma uniformly yellowish-brown with some irregular patches.

**Figure 1.**
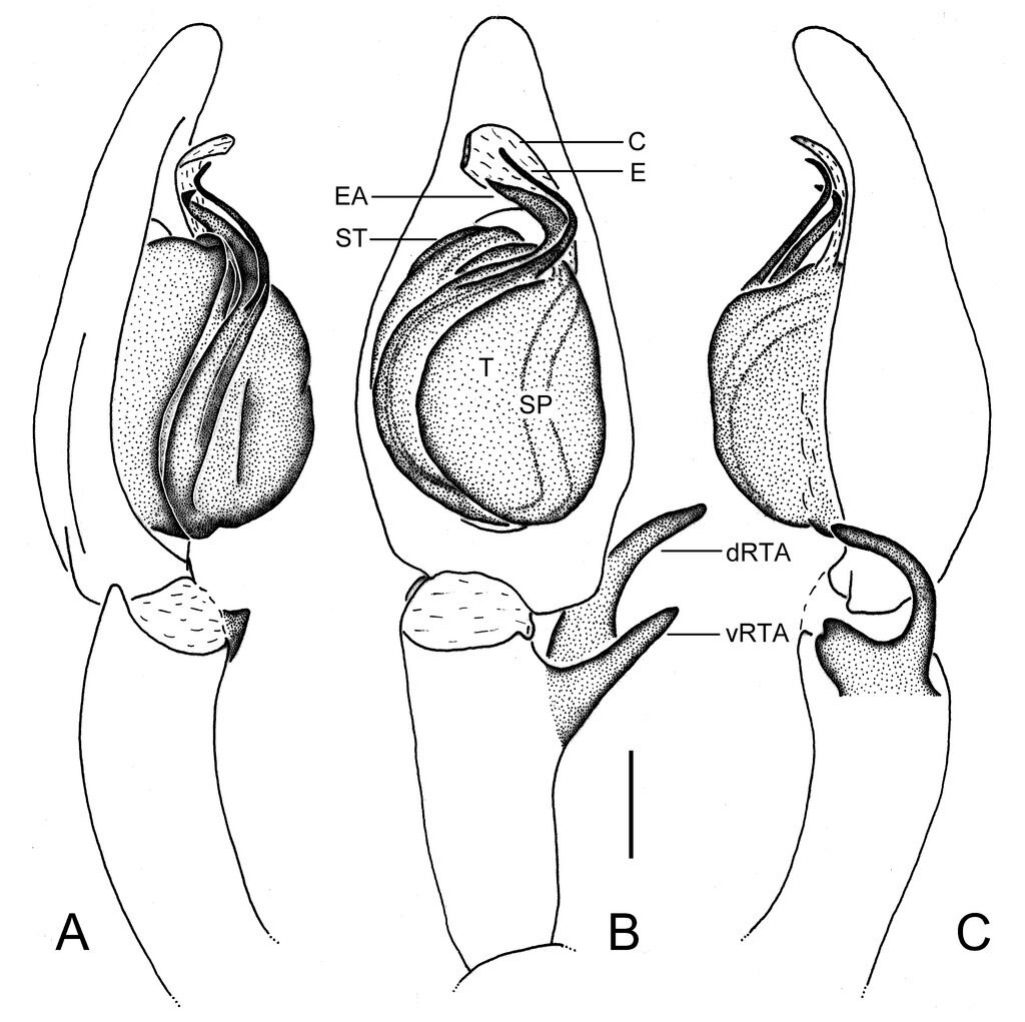
*Sinopoda angulata* Jäger, Gao & Fei, 2002, left male palp, prolateral (A), ventral (B) and retrolateral (C) view. Abbreviations: C—conductor, dRTA—dorsal retrolateral tibial apophysis, E—embolus, EA—embolic apophysis, SP—spermophore, ST—subtegulum, T—tegulum, vRTA—ventral retrolateral tibial apophysis. Scale bar: 0.5 mm.

**Figure 2.**
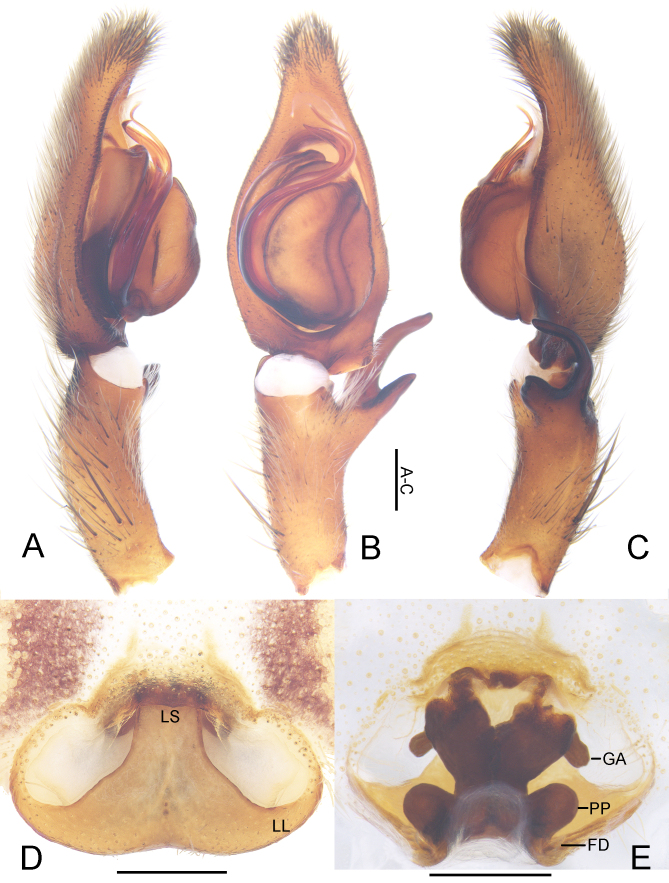
*Sinopoda angulata* Jäger, Gao & Fei, 2002. **A–C** Left male palp, prolateral (A), ventral (B) and retrolateral (C) view **D** Epigyne, ventral view **E** Vulva, dorsal view. Abbreviations: FD—fertilisation duct, GA—glandular appendage, LL—lateral lobes, LS—lobal septum, PP—posterior part of spermathecae. Scale bars: 0.5 mm.

**Figure 3.**
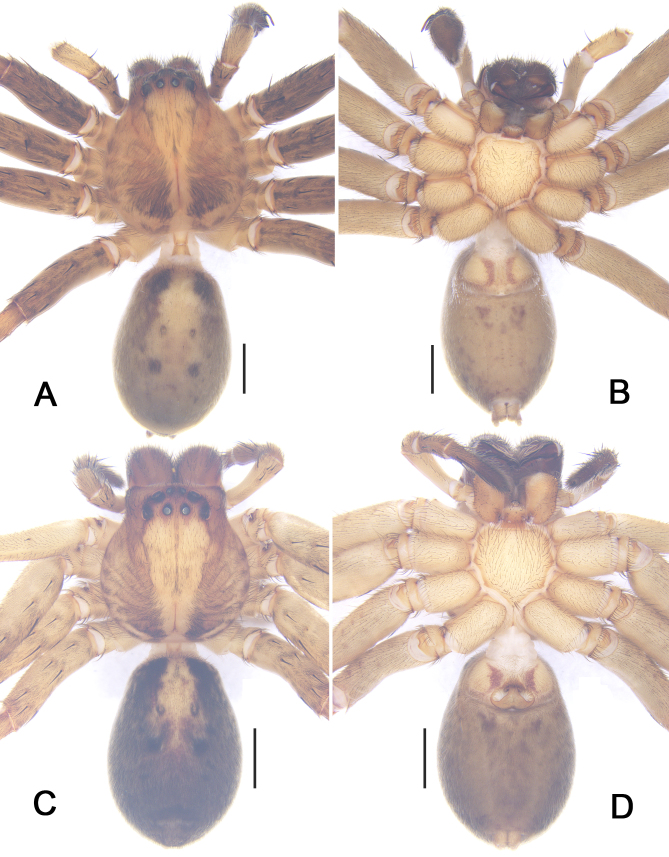
*Sinopoda angulata* Jäger, Gao & Fei, 2002. **A** male, dorsal view **B** male, ventral view **C** female, dorsal view **D** female, ventral view. Scale bars: 2 mm.

Palp as in diagnosis (Figs 1, 2). Cymbium longer than tibia. Conductor curved, arising in 1-o’clock position. Spermophore slightly curved in ventral view. Proximal part of tegulum covering embolus base. RTA arising subdistally from tibia with dRTA distinctly curved in retrolateral view.

***Female*.** For details see Jäger et al. 2002.

#### Diagnosis

Males of this species resemble those of *Sinopoda pyramidalis* Zhong, Jäger, Chen & Liu, 2019 (Zhong et al. 2019: Figs 50A–C and 51A–D) in having the embolus strongly S-shaped and embolic apophysis pointed in distal part, forming a triangle, but can be separated by: 1) embolus arising from tegulum at the 6-o’clock position in ventral view (8:30- to 9-o’clock position in *S. pyramidalis*); 2) dRTA narrow and dorsally digitiform in ventral view (broad, bulging in *S. pyramidalis*); 3) tegulum covering proximal part of embolus in ventral view (only small part of proximal embolus in *S. pyramidalis*) (Figs 1, 2A–C). Females of this species are similar to those of *Sinopoda shennonga* (Peng, Yin & Kim, 1996) (Jäger et al. 2002: Figs. 6–7) in having the lobal septum half the width of the lateral lobe and posterior part of spermathecae considerably larger than glandular projection, but differ from *S. shennonga* by: 1) epigynal field with distinct anterior bands (indistinct in *S. shennonga*); 2) epigyne with lobal pockets connected by an anterior rim (not in *S. shennonga*); 3) vulva with internal duct running parallel along the median line (diverging strongly anteriorly in *S. shennonga*). Females of this species can also be distinguished from other *Sinopoda* spp. by anterior vulva with a massive and angled structure (Fig. 2D–E, Jäger et al. 2002).

#### Distribution

China (Hubei Province) (Fig. 7).

### Sinopoda yichangensis sp. n.

1AC40971-7021-56AD-9756-DD42A9FF7B80

5F63E4F5-FEA8-422D-8FCA-B8C5274A1ABB

#### Materials

- **Type status:** Holotype. **Occurrence:** recordedBy: Jian Chen; individualCount: 1; sex: 1 male; lifeStage: adult; preparations: in ethyl alcohol; **Taxon:** order: Araneae; family: Sparassdiae; genus: Sinopoda; **Location:** country: China; countryCode: CHN; stateProvince: Hubei Province; county: Wufeng; decimalLatitude: 30.37; decimalLongitude: 110.54; **Identification:** identifiedBy: Jian Chen; **Event:** samplingProtocol: by hand; year: 2019; month: 8; day: 22

#### Description

***Male*** (Fig. 6). PL 6.5, PW 5.8, AW 2.7, OL 7.6, OW 4.0. Eyes: AME 0.29, ALE 0.45, PME 0.33, PLE 0.42, AME–AME 0.24, AME–ALE 0.10, PME–PME 0.51, PME–PLE 0.60, AME–PME 0.50, ALE–PLE 0.52, CH AME 0.15, CH ALE 0.20. Spination: Palp: 131, 101, 2121; Fe: I–III 323, IV 321; Pa: I–IV 000; Ti: I–III 2026, IV 2326; Mt: I–II 1014, III–IV 3036. Measurements of palp and legs: Palp 13.1 (3.7, 2.4, 2.8, –, 4.2), I 35.2 (8.8, 3.2, 9.5, 10.5, 3.2), II 37.5 (9.6, 2.9, 10.5, 11.3, 3.2), III 28.5 (8.1, 2.8, 7.4, 7.7, 2.5), IV 31.0 (8.5, 2.4, 8.1, 9.1, 2.9). Leg formula: II-I-IV-III. Cheliceral furrow with three anterior and four posterior teeth, with 31 denticles. Dorsal prosoma yellowish-brown, with distinct fovea and radial furrows. Chelicerae deep reddish-brown. Sternum reddish to yellowish-brown, with margin brown. Gnathocoxae and labium yellowish-brown, with distal parts lighter. Legs deep yellowish-brown, covered by short spines. Dorsal opisthosoma yellowish-purple, lateral field of opisthosoma with three pairs of black patches. Ventral opisthosoma deep yellowish-brown with irregular patches.

**Figure 4.**
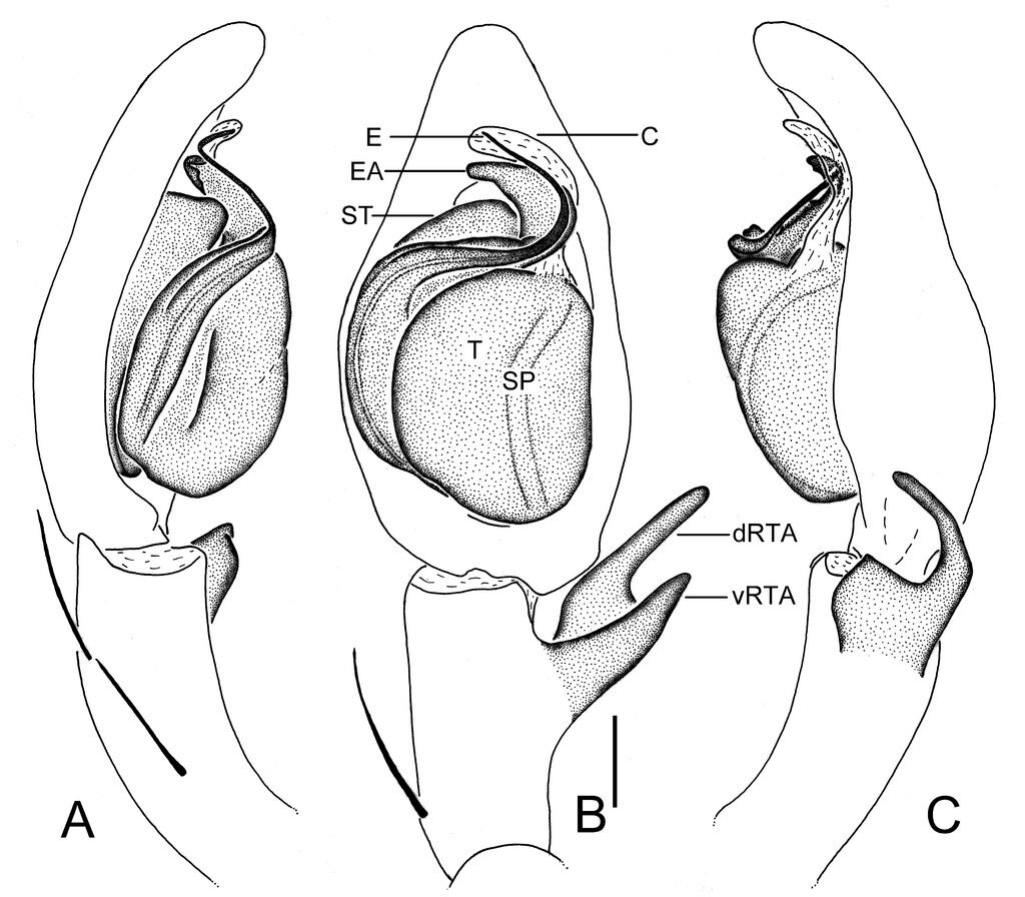
*Sinopoda yichangensis* sp. n., left male palp in prolateral (A), ventral (B) and retrolateral (C) view. Abbreviations: C—conductor, dRTA—dorsal retrolateral tibial apophysis, E—embolus, EA—embolic apophysis, SP—spermophore, ST—subtegulum, T—tegulum, vRTA—ventral retrolateral tibial apophysis. Scale bar: 0.5 mm.

**Figure 5.**
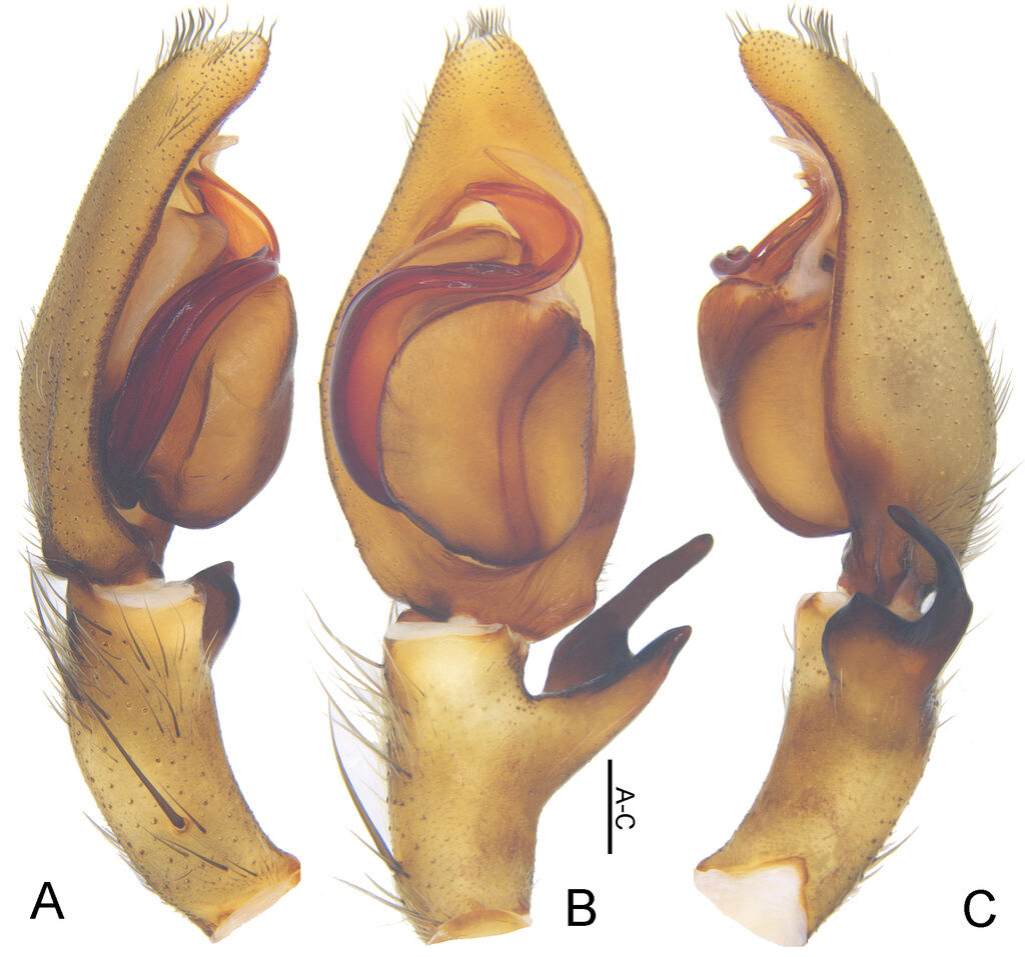
*Sinopoda yichangensis* sp. n., left male palp in prolateral (A), ventral (B) and retrolateral (C) view. Scale bar：0.5mm.

**Figure 6.**
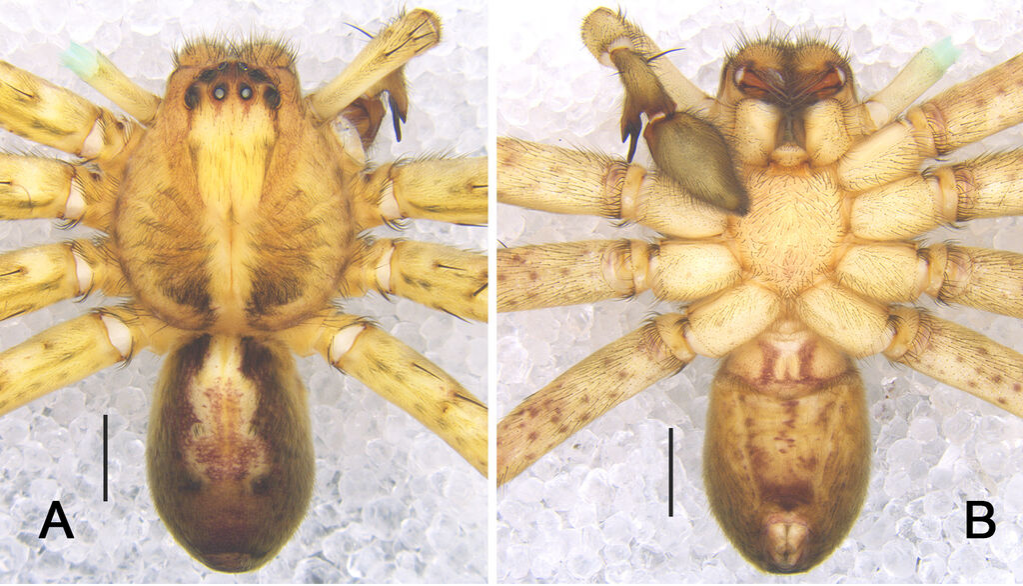
*Sinopoda yichangensis* sp. n., male in dorsal view (A) ventral view (B). Scale bars: 2 mm.

Palp as in diagnosis (Figs 4, 5). Cymbium about two times longer than tibia in ventral view. Conductor arising from tegulum in 1-o’clock-position. Tegulum median part slightly wider than proximal part. Spermophore slightly curved in ventral view. RTA arising subdistally from tibia, dRTA dorsally digitiform in ventral view and about two times as long as vRTA in retrolateral view.

***Female*.** Unknown.

#### Diagnosis

Males of this new species can be distinguished from all other *Sinopoda* species except *S. angulata* and *S. pyramidalis* (Zhong et al. 2019: Figs 50A–C and 51A–D) in having a thin embolus, as long as embolic apophysis, and embolic apohysis distinctly wider than embolus. It can be distinguished from the other two congeners by the following combination of characters: 1) tip of embolus apohysis with blunt ends (pointed ends in *S. angulata* and *S. pyramidalis*); 2) embolus arising from tegulum at 7- to 8-o’clock position in ventral view (6-o’clock position in *S. angulata*; 8:30- to 9-o’clock position in *S. pyramidalis*); 3) dRTA is straight with blunt ends in ventral view (slightly curved, with pointed ends in *S. angulata*; broad, bulging in *S. pyramidalis*) (Figs 1, 4).

#### Etymology

The specific name refers to the type locality; adjective.

#### Distribution

**Figure 7.**
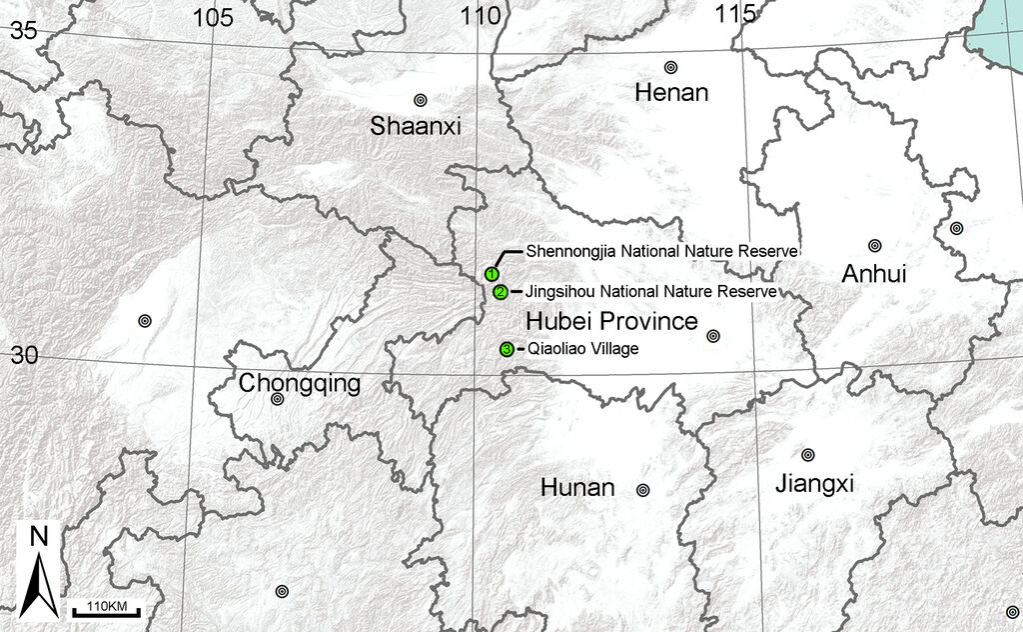
Locality records for two species of *Sinopoda*: 1, 2. *Sinopoda angulata* Jäger, Gao & Fei, 2002; 3. *Sinopoda yichangensis* sp. n.

Known only from the type locality (Fig. 7).

## Supplementary Material

XML Treatment for Sinopoda angulata

XML Treatment for Sinopoda yichangensis
